# Supplementary material for: Direct photoresponsive inhibition of a p53-like transcription activation domain in PIF3 by Arabidopsis phytochrome B
Source: Nat Commun. 2021 Sep 23;12:5614. doi: 10.1038/s41467-021-25909-5 (PMC8460787; doi:10.1038/s41467-021-25909-5)
Supplement: Supplementary file 1 — Supplementary Information [file 41467_2021_25909_MOESM1_ESM.pdf]

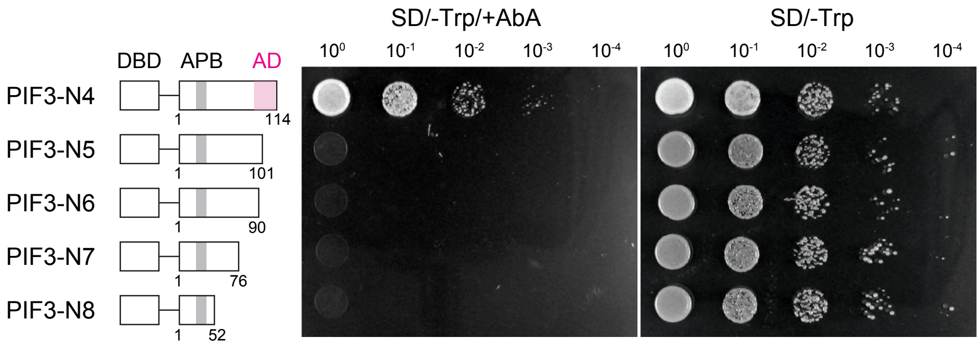

Supplementary Fig. 1. The aa$_{1-90}$ region does not exhibit transactivation activity in yeast. A series of N-terminal fragments of PIF3 between amino acids 1 and 101 were fused with Gal4-DBD as shown in the schematics and examined for their self-activation activity in yeast. Serial dilutions of the yeast strains containing the respective constructs were grown on either SD/-Trp/+AbA or SD/-Trp (control) media.

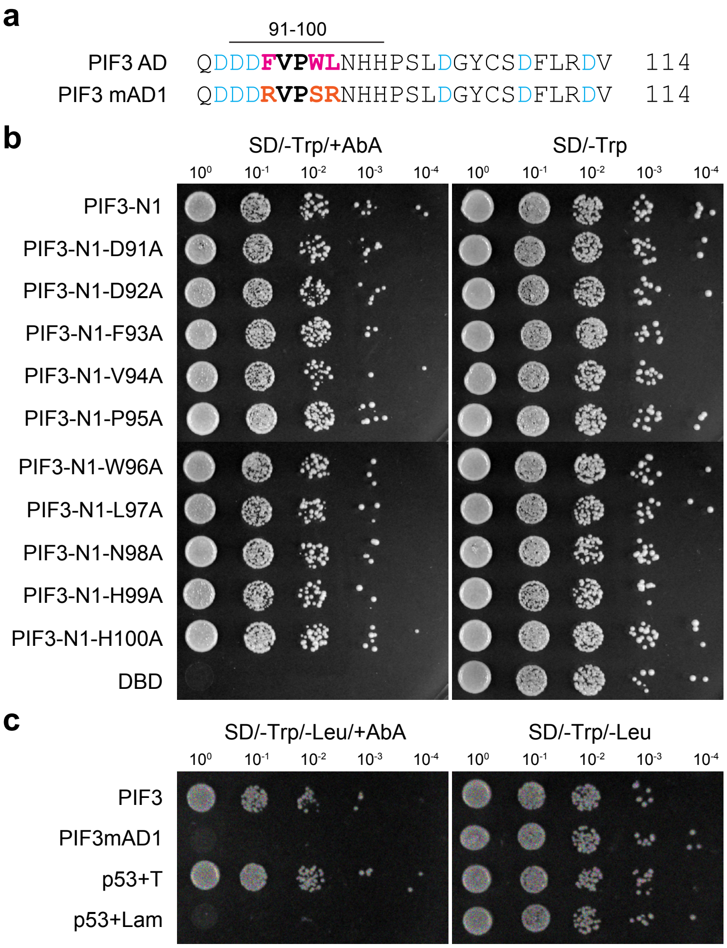

Suplementary Fig. 2. Abolishing the transactivation activity of PIF3 requires multiple mutations in the AD. a Sequence alignment of the PIF3 AD and the mAD1 mutant, in which the three hydrophobic residues in the ΦxxΦΦ activator motif were replaced with either an arginine or a serine (labeled in orange). The black bar indicates the region of amino acids 91 to 100, containing the conserved activator motif. **b** Yeast transactivation assays of DBD-PIF3-N1 (PIF3-N1) and DBD-PIF3-N1 mutants with individual amino acids between 91 and 100 replaced with an alanine. The yeast strains containing the respective constructs were grown on either SD/-Trp/+AbA or SD/-Trp (control) media. **c** Yeast transactivation assays of DBD-PIF3 (PIF3) and DBD-PIF3mAD1 (PIF3mAD1). Serial dilutions of the yeast strains containing the respective constructs were grown on either SD/-Trp/-Leu/+AbA or SD/-Trp/-Leu (control) media. Yeast strains containing p53 and either the SV40 large T-antigen (p53+T) or lamin (p53+Lam) were used as positive and negative controls, respectively.

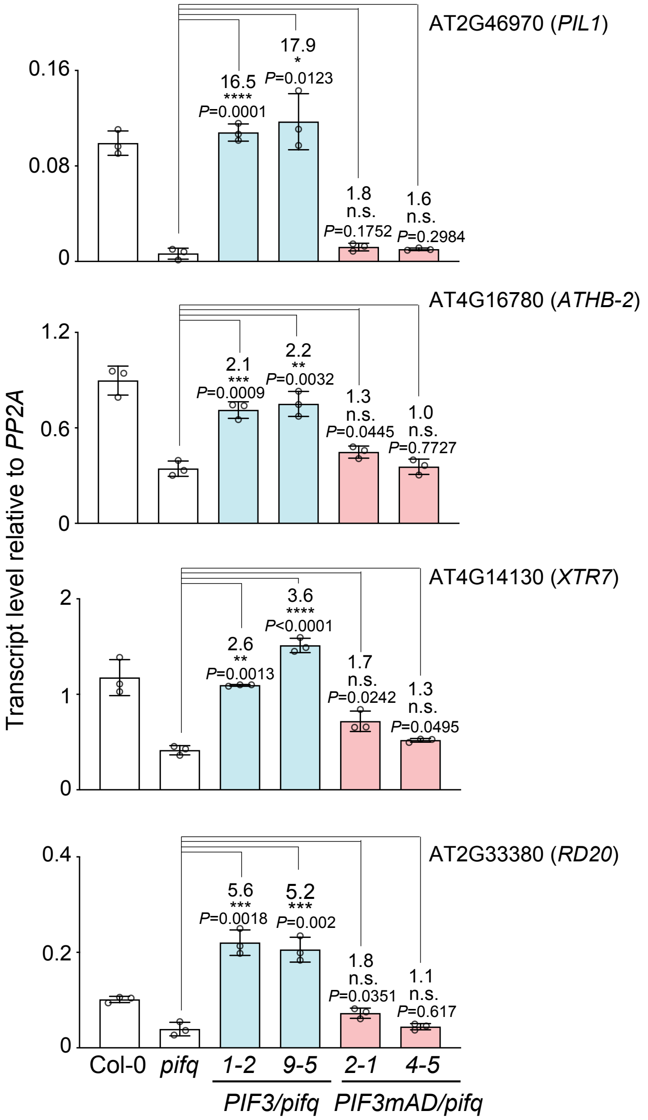

Supplementary Fig. 3. *PIF3mAD/pifq* lines exhibit reduced activity in PIF target gene expression in R light. qRT-PCR results showing the steady-state transcript levels of select PIF3 target genes in 4-d-old R-light-grown seedlings of Col-0, *pifq*, and the *PIF3/pifq* and *PIF3mAD/pifq* lines. The transcript levels were calculated relative to those of *PP2A*. Error bars represent the s.d. of three biological replicates. Numbers indicate fold changes relative to *pifq*; the statistical significance was analyzed using two-tailed Student's t-test (** $P \leq 0.01$, *** $P \leq 0.001$, **** $P \leq 0.0001$); n.s. indicates the difference is either less than 2-fold or not statistically significant.

Supplementary Table 1. PCR primers used for making the constructs of PIF3 and PIF3 paralogs.

	Insert	Vector	Forward primer	Reverse primer
pBridge-PIF3	*PIF3*	pBridge	acagttgactgtatcgccggctatgcctctgtttgagcttttcagg	ggaattagcttggctgcaggtcacgacgatccacaaaactg
pBridge-PIF3-N1	*PIF3-N1*	pBridge	acagttgactgtatcgccggctatgcctctgtttgagcttttcagg	ggaattagcttggctgcaggtcaacctgcttcctttcttccatc
pBridge-PIF3-N2	*PIF3-N2*	pBridge	acagttgactgtatcgccggctatgcctctgtttgagcttttcagg	ggaattagcttggctgcaggttacgggctttgaggactcttttc
pBridge-PIF3-N3	*PIF3-N3*	pBridge	acagttgactgtatcgccggctatgcctctgtttgagcttttcagg	ggaattagcttggctgcaggttacggattggtttgttggctagg
pBridge-PIF3-N4	*PIF3-N4*	pBridge	acagttgactgtatcgccggctatgcctctgtttgagcttttcagg	ggaattagcttggctgcaggttagggatgatgattcaaccatgg
pBridge-PIF3-N5	*PIF3-N5*	pBridge	acagttgactgtatcgccggctatgcctctgtttgagcttttcagg	ggaattagcttggctgcaggtcagggatgatgattcaaccat
pBridge-PIF3-N6	*PIF3-N6*	pBridge	acagttgactgtatcgccggctatgcctctgtttgagcttttcagg	ggaattagcttggctgcaggtcagtcttgactcaaacccgtc
pBridge-PIF3-N7	*PIF3-N7*	pBridge	acagttgactgtatcgccggctatgcctctgtttgagcttttcagg	ggaattagcttggctgcaggtcagatctcgtccaccatagtc
pBridge-PIF3-N8	*PIF3-N8*	pBridge	acagttgactgtatcgccggctatgcctctgtttgagcttttcagg	ggaattagcttggctgcaggtcaaggaatgttcctcgatcta
pBridge-PIF3-C1	*PIF3-C1*	pBridge	acagttgactgtatcgccggctatccctatgtcagtgccatc	ggaattagcttggctgcaggtcacgacgatccacaaaactg
pBridge-PIF3-C2	*PIF3-C2*	pBridge	acagttgactgtatcgccggctgatgactttgttccatggt	ggaattagcttggctgcaggtcacgacgatccacaaaactg
pBridge-PIF3-C3	*PIF3-C3*	pBridge	acagttgactgtatcgccggctccctcccttgatggatatt	ggaattagcttggctgcaggtcacgacgatccacaaaactg
pBridge-PIF3-M1	*PIF3-M1*	pBridge	acagttgactgtatcgccggctatccctatgtcagtgccatc	ggaattagcttggctgcaggttagggatgatgattcaaccatgg
pBridge-PIF3-M2	*PIF3-M2*	pBridge	acagttgactgtatcgccggctgatgactttgttccatggt	ggaattagcttggctgcaggttagggatgatgattcaaccatgg
pET42b-GST-PIF3	*PIF3*	pET42b	ggcgaattctatgcctctgtttgagcttttcag	ccgctcgagcgacgatccacaaaactgatc
pJHA212G-3HA-YFP-PIF3	*PIF3pro*	pJHA212 G-RBCSt	catgattacgaattcgagctataaaccagaagatgcaac	ggtgttgcgttttacagaa
	3HA-YFP		gtaaaacgcaacaccatgtacccatacgatgttc	gaaaagctcaaacagaggcataggggtgggagttggtgt
	PIF3		atgcctctgtttgagcttt	gcaggtcgactctagagtcacgacgatccacaaaac
pJHA212G-3HA-YFP-PIF3mAD	*PIF3pro*	pJHA212 G-RBCSt	catgattacgaattcgagctataaaccagaagatgcaac	ggtgttgcgttttacagaa
	3HA-YFP		gtaaaacgcaacaccatgtacccatacgatgttc	gaaaagctcaaacagaggcataggggtgggagttggtgt
	PIF3		atgcctctgtttgagcttt	gcaggtcgactctagagtcacgacgatccacaaaac
pBridge-PIF3/PHYB	*PIF3*	pBridge	acagttgactgtatcgccggctatgcctctgtttgagcttttcagg	ggaattagcttggctgcaggtcacgacgatccacaaaactg

pBridge-PIF3/PHYB	*PHYB-NLS*	pBridge	catccatacaatgggccatatggtttccggagtcggggg	agatcttcgggctaatgcttacacctttctcttcttcttaggatatggcatcatcagcat
pBridge-PIF3/PHYB-C	*PIF3*	pBridge	acagttgactgtatcgccggctatgcctctgtttgagcttttcagg	ggaattagcttggctgcaggtcacgacgatccacaaaactg
	PHYB-C-NLS		catccatacaatgggccatatgaactctaaagttgtcgat	agatcttcgggctaatgcttacacctttctcttcttcttaggatatggcatcatcagcat
pBridge-PIF3/NGB	*PIF3*	pBridge	acagttgactgtatcgccggctatgcctctgtttgagcttttcagg	ggaattagcttggctgcaggtcacgacgatccacaaaactg
	NGB		catccatacaatgggccatatggtttccggagtcggggg	aagatcttcgggctaatgcctatcctccaacctttctc
pBridge-PIF3/PHYA	*PIF3*	pBridge	acagttgactgtatcgccggctatgcctctgtttgagcttttcagg	ggaattagcttggctgcaggtcacgacgatccacaaaactg
	PHYA-NLS		catccatacaatgggccatatgtcaggctctaggccga	agatcttcgggctaatgcctaaacctttctcttcttcttaggcttgtttgctgcagcgag
pBridge-PIF1/PHYB	*PIF1*	pBridge	acagttgactgtatcgccggctatgcatcattttgtccctg	ggaattagcttggctgcaggttaacctgttgtgtggttt
	PHYB-NLS		catccatacaatgggccatatggtttccggagtcggggg	agatcttcgggctaatgcttacacctttctcttcttcttaggatatggcatcatcagcat
pBridge-PIF1/NGB	*PIF1*	pBridge	acagttgactgtatcgccggctatgcatcattttgtccctg	ggaattagcttggctgcaggttaacctgttgtgtggttt
	NGB		catccatacaatgggccatatggtttccggagtcggggg	aagatcttcgggctaatgcctatcctccaacctttctc
pBridge-PIF1/PHYA	*PIF1*	pBridge	acagttgactgtatcgccggctatgcatcattttgtccctg	ggaattagcttggctgcaggttaacctgttgtgtggttt
	PIF1/PHYA-NLS		catccatacaatgggccatatgtcaggctctaggccga	agatcttcgggctaatgcctaaacctttctcttcttcttaggcttgtttgctgcagcgag
pBridge-PIF1	*PIF1*	pBridge	acagttgactgtatcgccggctatgcatcattttgtccctg	ggaattagcttggctgcaggttaacctgttgtgtggttt
pBridge-PIF4	*PIF4*	pBridge	acagttgactgtatcgccggctatggaacaccaaggttgg	ggaattagcttggctgcaggctagtggtccaaacgaga
pBridge-PIF5	*PIF5*	pBridge	gactgtatcgccggctatggaacaagtgtttgctga	ttagcttggctgcaggtcagcctattttacccatat
pBridge-PIF7	*PIF7*	pBridge	gactgtatcgccggctatgtcgaattatggagttaa	ttagcttggctgcaggctaatctcttttctcatgat
pBridge-PIF8	*PIF8*	pBridge	gactgtatcgccggctatgtcccaatgtgttccaaa	ttagcttggctgcaggtcattttggattcgaaggaggag
pBridge-PIL1	*PIL1*	pBridge	ttgactgtatcgccggctatggaagcaaaacccttagc	ttagcttggctgcaggttagtttggcgagcgataat
pBridge-PIL2	*PIL2*	pBridge	gactgtatcgccggctatgatgttcttaccaaccga	ttagcttggctgcaggtcatctgttagttttccttg

Supplementary Table 2. Primers used for generating the mutant constructs of PIFs and PHYB.

Plasmid name	Gene name	Vector	Forward primer	Reverse primer
pBridge-PIF3-N1m1	*PIF3-N1m1*	pBridge	gagctggtgtgggaaaatggtc	ccattttcccacaccagctcagcggctgcggcagcaggtggagaagggttcctgtc
pBridge-PIF3-N1m2	*PIF3-N1m2*	pBridge	aatggtcagatatcaactcaaag	tgagttgatatctgaccattagcggctgcggcagccacaacttcatctacaggtgg
pBridge-PIF3-N1m3	*PIF3-N1m3*	pBridge	actcaaagtcagtcaagtagatc	ctacttgactgactttgagtagcggctgcggcagcttcccacaccagctccacaac
pBridge-PIF3-N1m4	*PIF3-N1m4*	pBridge	agtagatcgaggaacattcctc	ggaatgttcctcgatctactagcggctgcggcagctgatatctgaccattttcccac
pBridge-PIF3-N1m5	*PIF3-N1m5*	pBridge	attcctccaccacaagcaaac	tttgcttgtggtggaggaatagcggctgcggcagctgactgactttgagttgatatc
pBridge-PIF3-N1m6	*PIF3-N1m6*	pBridge	gcaaactcttctagagctagag	ctagctctagaagagtttgcagcggctgcggcagcgttcctcgatctacttgactg
pBridge-PIF3-N1m7	*PIF3-N1m7*	pBridge	gctagagagattggaaatggc	ccatttccaatctctctagcagcggctgcggcagcttgtggtggaggaatgttcctc
pBridge-PIF3-N1m8	*PIF3-N1m8*	pBridge	aatggctcaaagacgactatg	atagtcgtctttgagccattagcggctgcggcagctctagaagagtttgcttgtgg
pBridge-PIF3-N1m9	*PIF3-N1m9*	pBridge	actatggtggacgagatccc	gggatctcgtccaccatagtagcggctgcggcagctccaatctctctagctctagaag
pBridge-PIF3-N1m10	*PIF3-N1m10*	pBridge	cttcagtggagactgatatgcc	gatggcactgacatagggatagcggctgcggcagccgtctttgagccatttccaatc
pBridge-PIF3-N1m11	*PIF3-N1m11*	pBridge	ccatcactaatgacgggtttg	caaacccgtcattagtgatggagcggctgcggcagcctcgtccaccatagtcgtctttg
pBridge-PIF3-N1m12	*PIF3-N1m12*	pBridge	ggtttgagtcaagacgatgac	tcatcgtcttgactcaaaccagcggctgcggcagccactgacatagggatctcgtc
pBridge-PIF3-N1m13	*PIF3-N1m13*	pBridge	gatgactttgttccatggttg	aaccatggaacaaagtcatcagcggctgcggcagccgtcattagtgatggcactgac
pBridge-PIF3-N1m14	*PIF3-N1m14*	pBridge	tggttgaatcatcatccctcc	gagggatgatgattcaaccaagcggctgcggcagcgtcttgactcaaacccgtcattag
pBridge-PIF3-N1m15	*PIF3-N1m15*	pBridge	ccctcccttgatggatattgc	caatatccatcaagggagggagcggctgcggcagctggaacaaagtcatcgtcttg
pBridge-PIF3-N1m16	*PIF3-N1m16*	pBridge	tattgctctgatttcttgcg	gcaagaaatcagagcaatatgcagcagcggcggcatgatgattcaaccatggaa
pBridge-PIF3-N1m17	*PIF3-N1m17*	pBridge	ttgcgtgatgtgtcgtctc	gacgacacatcacgcaaggcagcagcggcagctccatcaagggagggatg
pBridge-PIF3-N1m18	*PIF3-N1m18*	pBridge	tcgtctcctgttactgtca	gacagtaacaggagacgacgcagcagccgcgaaatcagagcaatatccatc
pBridge-PIF3-N1-D91A	*PIF3-N1-D91A*	pBridge	agtcaagacgctgactttgttccatggttga	aacaaagtcagcgtcttgactcaaacccg

pBridge-PIF3-N1-D92A	*PIF3-N1-D92A*	pBridge	caagacgatgcctttgttccatggttgaatc	tggaacaaaggcatcgtcttgactcaaacc
pBridge-PIF3-N1-F93A	*PIF3-N1-F93A*	pBridge	agacgatgacgctgttccatggttgaatcat	ccatggaacagcgtcatcgtcttgactcaa
pBridge-PIF3-N1-V94A	*PIF3-N1-V94A*	pBridge	gatgactttgctccatggttgaatcatcat	caaccatggagcaaagtcatcgtcttgactc
pBridge-PIF3-N1-P95A	*PIF3-N1-P95A*	pBridge	tgactttgttgcatggttgaatcatcatc	attcaaccatgcaacaaagtcatcgtcttg
pBridge-PIF3-N1-W96A	*PIF3-N1-W96A*	pBridge	caagacgatgactttgttccagcattgaatcatcatccctcccttg	caagggagggatgatgattcaatgctggaacaaagtcatcgtcttg
pBridge-PIF3-N1-L97A	*PIF3-N1-L97A*	pBridge	gacgatgactttgttccatgggcaaatcatcatccctcccttgatg	catcaagggagggatgatgatttgcccatggaacaaagtcatcgtc
pBridge-PIF3-N1-N98A	*PIF3-N1-N98A*	pBridge	gatgactttgttccatggttggcacatcatccctcccttgatgg	ccatcaagggagggatgatgtgccaaccatggaacaaagtcatc
pBridge-PIF3-N1-H99A	*PIF3-N1-H99A*	pBridge	gactttgttccatggttgaatgcacatccctcccttgatggatattg	caatatccatcaagggagggatgtgcattcaaccatggaacaaagtc
pBridge-PIF3-N1-H100A	*PIF3-N1-H100A*	pBridge	ctttgttccatggttgaatcatgcaccctcccttgatggatattg	caatatccatcaagggagggtgcatgattcaaccatggaacaaag
pBridge-PIF3mAD	*PIF3 mAD*	pBridge	gctgctgcagcggcgaatcatcatccctccc	cgccgctgcagcagcgtcatcgtcttgactcaa
pBridge-PIF3/NGB-R110Q	*NGB-R110Q*	pBridge	aatccagcaaggtggttacattcagcc	tgtaaccaccttgctggattcgagagagat
pBridge-PIF3/NGB-G111D	*NGB-G111D*	pBridge	tccagcgagatggttacattcagcctttc	gaggagccttaagagtagaaccaaccaag
pBridge-PIF3/NGB-R352K	*NGB-R352K*	pBridge	tctactcttaaggctcctcatggttgtca	gaggagccttaagagtagaaccaaccaag
pBridge-PIF3mAD1	*PIF3mAD1*	pBridge	cgtgttccatctcgtaatcatcatccctcccttgatgga	atgattacgagatggaacacggtcatcgtcttgaca
pBridge-PIF1mAD	*PIF1mAD*	pBridge	gatgaagctgcggcggctgcgcattatcctctccgt	gataatgcgcagccgccgcagcttcatcttcttgaata
pBridge-PIF4mAD	*PIF4mAD*	pBridge	atcaagaagctgcggcggctgcgcaataccctccagatgaag	gtattgcgcagccgccgcagcttcttgatcttcaagaaag
pBridge-PIF5mAD	*PIF5mAD*	pBridge	caagaagctgcggcggctgcgcaatacctccggatgacgtc	agggtattgcgcagccgccgcagcttcttgatcatctag

pBridge-PIF7mAD	*PIF7mAD*	pBridge	cacctcggctgcggcggctgcgactcaaagtctcaacggtt	gactttgagtcgcagccgccgcagccgaggtggttggt
pBridge-PIF8mAD	*PIF8mAD*	pBridge	aggctgcggcggctgcgcatcatcgctcctcca	atgcgcagccgccgcagcctcatccgtgg

Supplementary Table 3. Primers for qRT-PCR analyses.

Accession	Gene name	Forward primer	Reverse primer
AT1G69960	*PP2A*	TATCGGATGACGATTCTTCGTGCAG	GCTTGGTCGACTATCGAATGAGAG
AT2G46970	*PIL1*	AAATTGCTCTCAGCCATTCGTGG	TTCTAAGTTTGAGGCGGACGCAG
AT4G16780	*ATHB2*	TCACAGTACTCTCAATCCGAAGC	CCGTAAGAACTCGCAGTCTAC
AT4G14130	*XTR7*	CACCGTCACTGCTTACTACTTG	CATTGGTGTGAAGAACATAAG
AT4G32280	*IAA29*	CACCATCATTGCCCGTATCA	CCACAGTAGCCGTTGTTGGA
AT2G33380	*RD20*	AAGGACGAAGATGGTTTCCTATC	CGAGAATTGGCCCTCTCTTT
AT4G14690	*ELIP2*	GGCAGAGGCAAAGTCAAAAGG	CGCAACGAGACCGAGCAT

Supplementary Table 4. Primer sets for ChIP-qPCR analysis of the *PIL1* locus.

Primer set	Forward primer	Reverse primer
1	ATGAATCACGCGGCATTC	ACGTGAGCGGAAAGAACC
2	GGATGAACAATGCACCACCAC	ACACGAAGGCACCACGAATG
3	CTCTATGACAGGAACATCACACC	ACAATGACTTGCCTTGTTTACAG
4	TCGAAGCAAAACCAATCCAAAC	GAATTGTGACCATTCTTTGTTCAG
5	AGTTGCATTATTGTTGGAGCATC	AAGGTCAGGAAAACACAATGTAG
6	CTGTGTCATAATCACGATTCAAGG	TTGGGGTTAATGAAGAGCAGC
