# Supplementary material for: Direct photoresponsive inhibition of a p53-like transcription activation domain in PIF3 by Arabidopsis phytochrome B
Source: Nat Commun. 2021 Sep 23;12:5614. doi: 10.1038/s41467-021-25909-5 (PMC8460787; doi:10.1038/s41467-021-25909-5)
Supplement: Supplementary file 3 — Reporting Summary [file 41467_2021_25909_MOESM3_ESM.pdf]

nature portfolio

Corresponding author(s): Meng Chen
Last updated by author(s): Sep 3, 2021

Reporting Summary

Nature Portfolio wishes to improve the reproducibility of the work that we publish. This form provides structure for consistency and transparency in reporting. For further information on Nature Portfolio policies, see our Editorial Policies and the Editorial Policy Checklist.

Statistics

For all statistical analyses, confirm that the following items are present in the figure legend, table legend, main text, or Methods section.

n/a	Confirmed	
☐	☒	The exact sample size (*n*) for each experimental group/condition, given as a discrete number and unit of measurement
☐	☒	A statement on whether measurements were taken from distinct samples or whether the same sample was measured repeatedly
☐	☒	The statistical test(s) used AND whether they are one- or two-sided<br>*Only common tests should be described solely by name; describe more complex techniques in the Methods section.*
☐	☒	A description of all covariates tested
☒	☐	A description of any assumptions or corrections, such as tests of normality and adjustment for multiple comparisons
☐	☒	A full description of the statistical parameters including central tendency (e.g. means) or other basic estimates (e.g. regression coefficient) AND variation (e.g. standard deviation) or associated estimates of uncertainty (e.g. confidence intervals)
☐	☒	For null hypothesis testing, the test statistic (e.g. *F*, *t*, *r*) with confidence intervals, effect sizes, degrees of freedom and *P* value noted<br>*Give P values as exact values whenever suitable.*
☒	☐	For Bayesian analysis, information on the choice of priors and Markov chain Monte Carlo settings
☒	☐	For hierarchical and complex designs, identification of the appropriate level for tests and full reporting of outcomes
☒	☐	Estimates of effect sizes (e.g. Cohen's *d*, Pearson's *r*), indicating how they were calculated

Our web collection on statistics for biologists contains articles on many of the points above.

Software and code

Policy information about availability of computer code

Data collection	N/A
Data analysis	ImageJ 1.8.0_172, GraphPad Prime 8, SpectraWiz v5.33, Adobe Creative Cloud 2021, Zeiss ZEN 2.3 imaging software

For manuscripts utilizing custom algorithms or software that are central to the research but not yet described in published literature, software must be made available to editors and reviewers. We strongly encourage code deposition in a community repository (e.g. GitHub). See the Nature Portfolio guidelines for submitting code & software for further information.

Data

Policy information about availability of data

All manuscripts must include a data availability statement. This statement should provide the following information, where applicable:

- Accession codes, unique identifiers, or web links for publicly available datasets
- A description of any restrictions on data availability
- For clinical datasets or third party data, please ensure that the statement adheres to our policy

Arabidopsis transgenic lines as well as plasmids generated during the current study are available from the corresponding author upon reasonable request. The source data underlying Figs 1a-b, 2c, 3, 4a-b, 4e-f, 5b-c, 6c-d, 7a-e, 8a-c, and Supplementary Fig. 3 are provided in a Source Data file.

Field-specific reporting

Please select the one below that is the best fit for your research. If you are not sure, read the appropriate sections before making your selection.

☒ Life sciences ☐ Behavioural & social sciences ☐ Ecological, evolutionary & environmental sciences

For a reference copy of the document with all sections, see nature.com/documents/nr-reporting-summary-flat.pdf

Life sciences study design

All studies must disclose on these points even when the disclosure is negative.

Sample size	No sample size calculation was performed. For the measurements of hypocotyl length, at least 20 seedlings were used.
Data exclusions	No data were excluded from analysis.
Replication	At least three biological replicates were performed for hypocotyl measurements, qRT-PCR analyses, pulldown assays, and immunoblots.
Randomization	Seedlings of wild-type, mutant, and transgenic lines were randomly picked for all analyses.
Blinding	Not relevant to this study.

Reporting for specific materials, systems and methods

We require information from authors about some types of materials, experimental systems and methods used in many studies. Here, indicate whether each material, system or method listed is relevant to your study. If you are not sure if a list item applies to your research, read the appropriate section before selecting a response.

Materials & experimental systems

n/a	Involved in the study	
☐	☒	Antibodies
☒	☐	Eukaryotic cell lines
☒	☐	Palaeontology and archaeology
☒	☐	Animals and other organisms
☒	☐	Human research participants
☒	☐	Clinical data
☒	☐	Dual use research of concern

Methods

n/a	Involved in the study	
☒	☐	ChIP-seq
☒	☐	Flow cytometry
☒	☐	MRI-based neuroimaging

Antibodies

Antibodies used	Anti-PIF3 antibody, anti-GFP antibody (Abcam, cat. no. ab290), anti-RPN6 antibody (Enzo Life Sciences, BML-PW8370-0100), anti-HA antibody (MilliporeSigma, H3663), and goat anti-rabbit secondary antibody (Bio-Rad, 1706515).
Validation	The anti-PIF3 antibody was reported previously and can bu validated using the pifq mutant (Chen et al. 2010 Cell 141:1230-40). The validation data for the rest of the antibodies can be found in the manufacturer's website.
